# Travel restrictions and SARS-CoV-2 transmission: an effective distance approach to estimate impact

**DOI:** 10.2471/BLT.20.255679

**Published:** 2020-05-28

**Authors:** Shoi Shi, Shiori Tanaka, Ryo Ueno, Stuart Gilmour, Yuta Tanoue, Takayuki Kawashima, Shuhei Nomura, Akifumi Eguchi, Hiroaki Miyata, Daisuke Yoneoka

**Affiliations:** a Graduate School of Medicine, University of Tokyo, Tokyo, Japan.; b Center for Public Health Sciences, National Cancer Center, Tokyo, Japan.; c The Australian and New Zealand Intensive Care Research Centre, Melbourne, Australia.; d Graduate School of Public Health, St Luke’s International University, 3-6-2 Tsukiji, Chuo-ku, Tokyo 104-0045, Japan.; e Institute for Business and Finance, Waseda University, Tokyo, Japan.; f Department of Mathematical and Computing Science, Tokyo Institute of Technology, Tokyo, Japan.; g Department of Health Policy and Management, Keio University, Tokyo, Japan.; h Center for Preventive Medical Sciences, Chiba University, Chiba, Japan.

## Abstract

**Objective:**

To estimate the effect of airline travel restrictions on the risk of severe acute respiratory syndrome coronavirus 2 (SARS-CoV-2) importation.

**Methods:**

We extracted passenger volume data for the entire global airline network, as well as the dates of the implementation of travel restrictions and the observation of the first case of coronavirus disease (COVID-19) in each country or territory, from publicly available sources. We calculated effective distance between every airport and the city of Wuhan, China. We modelled the risk of SARS-CoV-2 importation by estimating survival probability, expressing median time of importation as a function of effective distance. We calculated the relative change in importation risk under three different hypothetical scenarios that all resulted in different passenger volumes.

**Findings:**

We identified 28 countries with imported cases of COVID-19 as at 26 February 2020. The arrival time of the virus at these countries ranged from 39 to 80 days since identification of the first case in Wuhan. Our analysis of relative change in risk indicated that strategies of reducing global passenger volume and imposing travel restrictions at a further 10 hub airports would be equally effective in reducing the risk of importation of SARS-CoV-2; however, this reduction is very limited with a close-to-zero median relative change in risk.

**Conclusion:**

The hypothetical variations in observed travel restrictions were not sufficient to prevent the global spread of SARS-CoV-2; further research should also consider travel by land and sea. Our study highlights the importance of strengthening local capacities for disease monitoring and control.

## Résumé

**Objectif:**

Évaluer les effets des restrictions de voyage par voie aérienne sur le risque d'importer le coronavirus 2 du syndrome respiratoire aigu sévère (SARS-CoV-2).

**Méthodes:**

Nous avons prélevé les données sur le volume de passagers pour l'ensemble du réseau aérien dans le monde, ainsi que les dates de mise en œuvre des restrictions de voyage et l'observation du premier cas de maladie à coronavirus (COVID-19) dans chaque pays ou territoire, à partir de sources accessibles au public. Nous avons calculé la distance réelle entre chaque aéroport et la ville de Wuhan, en Chine. Nous avons modélisé le risque d'importation du SARS-CoV-2 en estimant sa probabilité de survie, la période médiane d'importation étant exprimée en fonction de la distance réelle. Nous avons déterminé l'évolution relative du risque d'importation selon trois scénarios hypothétiques, qui ont tous débouché sur différents volumes de passagers.

**Résultats:**

Nous avons identifié 28 pays ayant importé des cas de COVID-19 au 26 février 2020. La date d'arrivée du virus dans ces pays s'est étalée sur une période de 39 à 80 jours depuis la détection du premier cas à Wuhan. Notre analyse de l'évolution relative du risque a indiqué que les stratégies de réduction du nombre global de passagers et de restriction des voyages dans 10 aéroports de première catégorie auraient eu le même niveau d'efficacité dans la diminution du risque d'importation du SARS-CoV-2. Néanmoins, cette diminution est très limitée, avec une évolution relative médiane du risque proche de zéro.

**Conclusion:**

Les variations hypothétiques présentes dans les restrictions de voyage observées n'étaient pas suffisantes pour éviter la propagation du SARS-CoV-2 aux quatre coins de la planète. Les futures recherches à ce propos devraient également tenir compte des déplacements par voie terrestre et maritime. Notre étude souligne l'importance de renforcer les capacités locales de surveillance et de contrôle des maladies.****

## Resumen

**Objetivo:**

Estimar el efecto de las restricciones a los viajes en avión sobre el riesgo de importación del coronavirus tipo 2 del síndrome respiratorio agudo grave (SARS-CoV-2).

**Métodos:**

Se obtuvieron los datos sobre el volumen de pasajeros de toda la red global de aerolíneas, así como las fechas en las que se implementaron las restricciones de viaje y la observación del primer caso de la enfermedad del coronavirus (COVID-19) en cada país o territorio, a partir de fuentes a disposición del público. Se calculó la distancia efectiva entre cada aeropuerto y la ciudad de Wuhan, China. Se diseñó un modelo del riesgo de importación del SARS-CoV-2 al estimar la probabilidad de supervivencia, que indica el tiempo medio de importación en función de la distancia efectiva. Se calculó el cambio relativo en el riesgo de importación bajo tres escenarios hipotéticos diferentes que arrojaron valores distintos sobre el volumen de pasajeros.

**Resultados:**

Se identificaron 28 países con casos importados de la COVID-19 al 26 de febrero de 2020. El tiempo de llegada del virus a estos países osciló entre 39 y 80 días desde la identificación del primer caso en Wuhan. El análisis que se expone aquí sobre el cambio relativo del riesgo indicó que las estrategias orientadas a reducir el volumen de pasajeros a nivel mundial y a imponer restricciones a los viajes en otros 10 aeropuertos centrales serían igualmente efectivas para reducir el riesgo de importación del SARS-CoV-2; sin embargo, esta reducción es muy limitada si se tiene en cuenta que el cambio relativo medio del riesgo es casi nulo.

**Conclusión:**

Las variaciones hipotéticas de las restricciones de viaje que se observaron no fueron suficientes para impedir la propagación global del SRAS-CoV-2; además, se debería analizar la posibilidad de viajar por tierra y por mar en las investigaciones futuras. Este estudio destaca la importancia de fortalecer la capacidad local para monitorear y controlar la enfermedad.

## ملخص

الغرض تقييم تأثير فرض قيود للسفر عبر الخطوط الجوية، على خطر جلب فيروس المتلازمة التنفسية الحادة الشديدة المعروفة باسم كورونا فيروس 2 (سارس كوف 2).

الطريقة قمنا باستخراج بيانات حجم الركاب لشبكة الخطوط الجوية العالمية بالكامل، بالإضافة إلى تواريخ تنفيذ قيود السفر، وملاحظة الحالة الأولى لمرض فيروس كورونا (كوفيد 19) في كل بلد أو إقليم، من المصادر المتاحة للجمهور. قمنا بحساب المسافة الفعالة بين كل مطار ومدينة ووهان، في الصين. وقمنا بوضع نموذج لخطر جلب سارس كوف 2 من خلال تقدير احتمالية البقاء، مع التعبير عن متوسط ​​وقت الجلب كدالة للمسافة الفعالة. واحتسبنا التغيير النسبي في مخاطر الجلب في ظل ثلاثة سيناريوهات افتراضية مختلفة أدت جميعها إلى أحجام مختلفة للركاب.

النتائج حددنا 28 دولة بها حالات تم جلبها من كوفيد 19 حتى 26 فبراير/شباط 2020. تراوح وقت وصول الفيروس في هذه البلدان من 39 إلى 80 يوماً منذ تحديد الحالة الأولى في ووهان. أشار تحليلنا للتغيير النسبي في المخاطر، إلى أن استراتيجيات الحد من حجم الركاب العالمي، وفرض قيود السفر في 10 مطارات مركزية أخرى، ستكون فعالة بنفس القدر في الحد من خطر جلب فيروس سارس كوف 2؛ إلا أن هذا الانخفاض في المخاطر محدود للغاية مع متوسط للتغيير النسبي يقترب من الصفر.

الاستنتاج لم تكن الاختلافات الافتراضية في قيود السفر الملحوظة كافية للحيلولة دون الانتشار العالمي لسارس كوف 2؛ ويجب أن تعتني المزيد من الأبحاث أيضا بالسفر البري وعبر البحر. تركز الدراسة الخاصة بنا على أهمية تعزيز القدرات المحلية لمكافحة المرض والسيطرة عليه.

## 摘要

**目的:**

旨在评估航空出行限制对严重急性呼吸综合征冠状病毒 2 (SARS-CoV-2) 输入风险的影响。

**方法:**

我们从公开来源中提取了全球所有航空公司网络的客运量数据，以及各个国家或地区实施出行限制的日期和观察到首例 2019 冠状病毒病 (COVID-19) 患者的日期。我们计算了每个机场和中国武汉之间的有效距离。我们通过估计存活概率来模拟严重急性呼吸综合征冠状病毒 2 (SARS-CoV-2) 的输入风险，将输入时间的中位数用一个含有效距离的函数式来表示。我们计算了三种不同假设情境下输入风险的相对变化，这三种情境下的客运量均不相同。

**结果:**

截至 2020 年 2 月 26 日，我们确定了 28 个 2019 冠状病毒病输入病例国家。自武汉确诊首例患者以来，病毒传播至这些国家的时间从 39 天到 80 天不等。我们对风险相对变化的分析表明，减少全球客运量和在另外 10 个枢纽机场实施出行限制的策略在降低严重急性呼吸综合征冠状病毒 2 (SARS-CoV-2) 输入风险方面有着同样的效果；然而，这种减少是非常有限的，因为风险的相对变化中值接近于零。

**结论:**

所观察到的出行限制的假设变化不足以防止严重急性呼吸综合征冠状病毒 2 (SARS-CoV-2) 在全球的传播；未来的研究还应该考虑陆路和海路出行。此项研究突出强调加强地方疾病监测和控制能力的重要性。

## Резюме

**Цель:**

Оценить влияние ограничения воздушного сообщения на риск завоза тяжелого острого коронавирусного респираторного синдрома (SARS-CoV-2).

**Методы:**

Авторы взяли из общедоступных источников данные об объеме пассажирских перевозок для всей международной сети авиалиний, а также датах ввода ограничений на путешествия и сроках выявления первых случаев коронавирусной болезни (COVID-19) в каждой отдельной стране или территории. Было рассчитано эффективное расстояние между каждым аэропортом и городом Ухань в Китае. Авторы смоделировали риск завоза вируса SARS-CoV-2 посредством оценки вероятности выживания, выразив медианное время завоза как функцию эффективного расстояния. Было рассчитано относительное изменение риска завоза инфекции для трех различных гипотетических сценариев, все из которых привели к разному объему пассажирского потока.

**Результаты:**

По состоянию на 26 февраля 2020 года было выявлено 28 стран, в которые была завезена инфекция, вызывающая COVID-19. Срок попадания вируса в эти страны составлял от 39 до 80 дней с момента выявления первого случая заболевания в Ухане. Анализ относительного изменения риска указывает на то, что стратегии сокращения глобального объема пассажирских перевозок и наложения ограничений на перелеты в 10 дополнительных крупных узловых аэропортах были бы так же эффективны для снижения риска завоза вируса SARS-CoV-2, однако такое снижение риска очень ограниченно и имеет близкое к нулю медианное значение относительного изменения риска.

**Вывод:**

Гипотетические вариации наблюдаемых ограничений воздушного сообщения не были достаточны для предотвращения глобального распространения SARS-CoV-2. В последующих исследованиях следует также учитывать поездки наземным транспортом и по морю. Исследование подчеркивает важность укрепления потенциала для мониторинга и контроля заболеваний на местах.

## Introduction

As of 25 May 2020, 347 697 deaths resulting from 5 392 654 laboratory-confirmed infectious cases of coronavirus disease (COVID-19) had been reported.^1^^,^^2^ Global travel contributed to the rapid growth of cases in Wuhan, China and internationally, including other Asian countries, Europe and the United States of America.^1^^,^^3^^,^^4^ Most of the current strategies to reduce the risk of severe acute respiratory syndrome coronavirus 2 (SARS-CoV-2) transmission are based on controlling interactions between humans, including case isolation, tracking patient contacts and screening air passengers crossing national borders.

International airline travellers departing from China have unintentionally transported SARS-CoV-2 all over the world. As of 30 January 2020, the Emergency Committee convened by the World Health Organization (WHO), acting according to International Health Regulations, described the unfolding outbreak as a Public Health Emergency of International Concern; the Committee proposed several recommendations to strengthen the monitoring and surveillance of the virus at an international level. Based on the available information at that time, the Committee did not recommend any restrictions on global travel or trade for any countries, including China.^5^ Nevertheless, as during previous infectious epidemics such as Ebola virus, SARS and influenza H1N1–2009, by 26 February 2020, at least 80 countries and territories had adopted travel restrictions in response to the threat of importation of SARS-CoV-2.

Reacting to the rapid dissemination of emerging infectious diseases by airline travel, researchers have developed several mathematical modelling techniques.^6^^–^^11^ In 2006, an overall review was published on the relationship between the global transportation network and the spread of infectious diseases.^12^ Later, researchers proposed a new stochastic meta-population model that incorporated travel records and census data in 220 countries to examine a prediction accuracy for the global spread of SARS.^11^ A large-scale simulation examined the impact of travel restrictions on the delay of the Ebola epidemic, demonstrating that restrictions delayed the outbreak of Ebola for approximately 30 days in African countries.^9^ Another study estimated the absolute (< 1%) and relative (~20%) reductions in risk due to travel restrictions, and concluded that such restrictions were not effective in preventing the global spread of Ebola.^10^ Here we follow the methods used in that study.^10^

It is still unclear how air travel restrictions contribute to the control of SARS-CoV-2 transmission.^4^ We therefore evaluate the effect of travel restrictions on the risk of SARS-CoV-2 transmission, using a hazard-based model and the concept of effective distance. We apply our model to travel network data to explore patterns of domestic and international population movement from Wuhan. 

## Methods

### Data set

For the 80 countries with a travel restriction in place by 26 February 2020 (Table 1), we extracted the dates of the introduction of travel restrictions and of the identification of the first case of COVID-19 from publicly available secondary data sources.^13^^,^^14^ To check the validity of these data sources, we confirmed all extracted dates with official announcements from individual governments. The travel restrictions were of the form: (i) entry or exit bans, defined as general restrictions on the ability of people to depart from their country for travel to China or the ability of foreigners to enter a country after travelling from or transiting via China; (ii) visa restrictions, defined as total or partial visa suspensions for travellers from China; or (iii) flight suspensions, defined as governmental bans on flights to or from China.^14^^,^^15^

**Table 1:** Dates of introduction of travel restrictions and first reported cases of COVID-19 as at 26 February 2020

Country or territory	Date of introduction of travel restriction	Date of first reported case of COVID-19 (category)^a^	Arrival time in days
Afghanistan	30 Jan	24 Feb (B)	78
Algeria	None	26 Feb (A)	80
Antigua and Barbuda	31 Jan	None (C)	NA
Armenia	1 Feb	None (C)	NA
Australia	1 Feb	25 Jan (A)	48
Austria	14 Feb	None (C)	NA
Azerbaijan	1 Feb	None (C)	NA
Bahamas	30 Jan	None (C)	NA
Bahrain	13 Feb	24 Feb (B)	78
Bangladesh	2 Feb	None (C)	NA
Belgium	None	6 Feb (A)	60
Belize	8 Feb	None (C)	NA
Brazil	None	26 Feb (A)	80
Brunei Darussalam	31 Jan	None (C)	NA
Cambodia	None	6 Feb (A)	60
Canada	None	27 Jan (A)	50
China, Macao SAR	28 Jan	None (C)	NA
China, Taiwan	7 Feb	None (C)	NA
China, Hong Kong SAR	27 Jan	None (C)	NA
Cook Islands	31 Jan	None (C)	NA
Croatia	None	26 Feb (A)	80
Czechia	9 Feb	None (C)	NA
Egypt	1 Feb	15 Feb (B)	69
El Salvador	31 Jan	None (C)	NA
Fiji	2 Feb	None (C)	NA
Finland	None	31 Jan (A)	54
France	30 Jan	None (C)	NA
French Polynesia	7 Feb	None (C)	NA
Gabon	7 Feb	None (C)	NA
Germany	30 Jan	28 Jan (A)	51
Grenada	2 Feb	None (C)	NA
Guatemala	31 Jan	None (C)	NA
Guyana	31 Jan	None (C)	NA
India	2 Feb	30 Jan (A)	53
Indonesia	2 Feb	None (C)	NA
Iran (Islamic Republic of)	31 Jan	None (C)	NA
Iraq	2 Feb	23 Feb (B)	77
Israel	2 Feb	23 Feb (B)	77
Italy^b^	31 Jan	None (C)	NA
Jamaica	31 Jan	None (C)	NA
Japan	1 Feb	16 Jan (A)	39
Jordan	5 Feb	None (C)	NA
Kazakhstan	3 Feb	None (C)	NA
Kenya	31 Jan	None (C)	NA
Kuwait	6 Feb	None (C)	NA
Kyrgyzstan	1 Feb	None (C)	NA
Lao People's Democratic Republic	2 Feb	None (C)	NA
Lebanon	None	22 Feb (A)	76
Madagascar	11 Feb	None (C)	NA
Malaysia	9 Feb	None (C)	NA
Maldives	3 Feb	None (C)	NA
Mauritius	2 Feb	None (C)	NA
Mongolia	6 Feb	None (C)	NA
Morocco	31 Jan	None (C)	NA
Mozambique	28 Jan	None (C)	NA
Nauru	4 Feb	None (C)	NA
Nepal	None	24 Jan (A)	47
New Zealand	2 Feb	None (C)	NA
Niue	3 Feb	None (C)	NA
Oman	3 Feb	None (C)	NA
Pakistan	None	27 Jan (A)	50
Palau	1 Feb	None (C)	NA
Papua New Guinea	29 Jan	None (C)	NA
Paraguay	31 Jan	None (C)	NA
Philippines	27 Jan	30 Jan (B)	NA
Qatar	3 Feb	None (C)	NA
Republic of Korea	4 Feb	20 Jan (A)	43
Russian Federation	31 Jan	None (C)	NA
Rwanda	30 Jan	None (C)	NA
Saint Kitts and Nevis	1 Feb	None (C)	NA
Saint Lucia	4 Feb	None (C)	NA
Samoa	4 Feb	None (C)	NA
Saudi Arabia	2 Feb	None (C)	NA
Seychelles	29 Jan	None (C)	NA
Singapore	29 Jan	23 Jan (A)	46
Spain	None	6 Feb (A)	60
Sri Lanka	28 Jan	28 Jan (A)	51
Suriname	5 Feb	None (C)	NA
Sweden	None	6 Feb (A)	60
Switzerland	30 Jan	25 Feb (B)	79
Tajikistan	30 Jan	None (C)	NA
Thailand	None	16 Jan (A)	39
Tonga	3 Feb	None (C)	NA
Trinidad and Tobago	30 Jan	None (C)	NA
Turkey	5 Feb	None (C)	NA
Turkmenistan	31 Jan	None (C)	NA
United Arab Emirates	5 Feb	None (C)	NA
United Kingdom	14 Feb	None (C)	NA
United Republic of Tanzania	29 Jan	None (C)	NA
United States	2 Feb	22 Jan (A)	45
Uzbekistan	1 Feb	None (C)	NA
Vanuatu	9 Feb	None (C)	NA
Viet Nam	1 Feb	None (C)	NA

COVID-19: coronavirus disease 2019; NA: not applicable; SAR: Special Administrative Region; SARS-CoV-2: severe acute respiratory syndrome coronavirus 2.

^a^ We categorized the 200 countries into three separate groups: A, those that imported SARS-CoV-2 before travel restrictions were put in place; B, those that imported SARS-CoV-2 after the introduction of travel restrictions; and C, those that had not imported the virus.

^b^ The date of the first case of COVID-19 in Italy was 31 January 2020; however, this information was not available at the time of our study.

We used Automatic Dependent Surveillance-Broadcast (ADS-B) exchange data to construct the airline transportation network encompassing the 200 countries and territories we included in this analysis.^16^^,^^17^ These publicly available exchange data are in the form of an airline network diagram consisting of 1773 nodes (i.e. airports) and 23 505 edges (i.e. direct flights), as of 1 December 2019. The weight of each edge represents passenger volume on a direct flight between two nodes. We estimated passenger volume on any particular flight by multiplying the number of seats available on the aeroplane by a factor of 0.7.^10^^,^^18^ The data and codes are available in the data repository.^17^

### Effective distance

We examined the impact of airline network restrictions on the transmission of SARS-CoV-2 by calculating effective distance^19^ from the network diagram. We did not consider the incubation period of the virus and all passengers were considered equal in terms of the number of people they could potentially infect. We calculated effective distance, defined as the minimum distance between each node where both path length (i.e. distance between a pair of nodes) and the degree of each node (i.e. the number of edges from the node) are taken into consideration, using the adjacency matrix of the network diagram (i.e. the square matrix whose elements indicate whether pairs of nodes are adjacent or not in the graph). Previous studies have demonstrated the ability of effective distance, as opposed to geographical distance, to predict the arrival time of a virus (i.e. the time between emergence at its source and its importation). For example, effective distance has successfully been used to predict the global spread of SARS and influenza H1N1–2009,^19^ to forecast in real time the spread of Middle East respiratory syndrome coronavirus (MERS-CoV) and Zika virus,^18^^,^^20^ and to determine the impact of travel restrictions on the spread of Ebola virus.^10^

Effective distance *d_ij_* between the *i*th airport in the *j*th country and Wuhan airport is defined as the minimum of all possible effective path lengths (i.e. path length with passenger volume considered). The effective path length from Wuhan city to the *i*th airport, with a sequence of *l* – 1 transit airports 
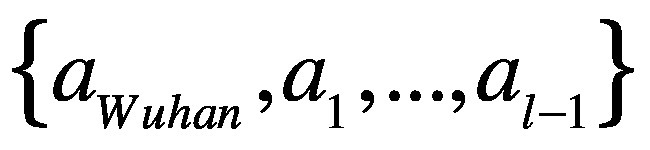
, is defined:^18^
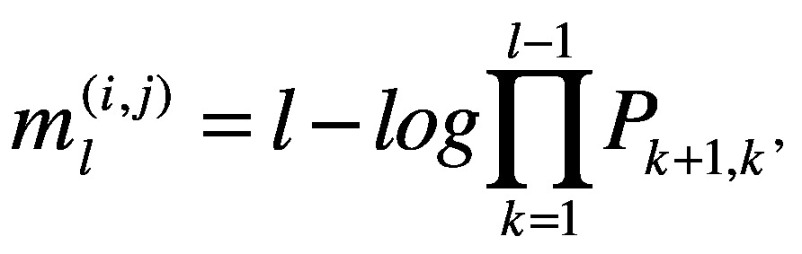
(1)Where *P_l,m_* denotes the conditional (transition) probability that any particular individual travelled from the *l*th to the *m*th airport.^18^ This transition probability is estimated as *P_l,m_ = w_lm_/Σ_n_w_ln_* where *w_lm_* is the passenger volume that travelled from the *l*th to the *m*th airport. To take into account the fact that the network diagram and the associated effective distances changed with the introduction of travel restrictions, we made the assumption that 75% of the number of direct flights were cancelled (where the number of direct flights that took place on 1 December 2019 is considered as 100%).^9^^,^^10^ We denote the effective distance before and after the travel restrictions 
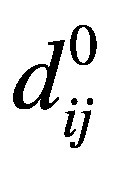
 and 
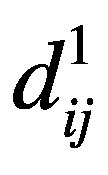
, respectively.

### Hazard-based model

We modelled the risk of importing SARS-CoV-2 by estimating survival probability. We define the survival probability as 
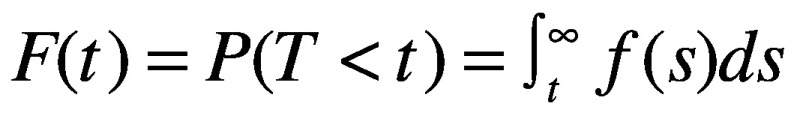
 with the probability density function *f(t)*, where *t = 0* corresponds to the date of observation of the first case of COVID-19 in Wuhan city (i.e. 8 December 2019) and *T* is a random (continuous) variable indicating the time from *t* = 0 to its importation at the *i*th airport in the *j*th country. The hazard function for virus importation for the *i*th airport in the *j*th country is defined:^10^^,^^19^
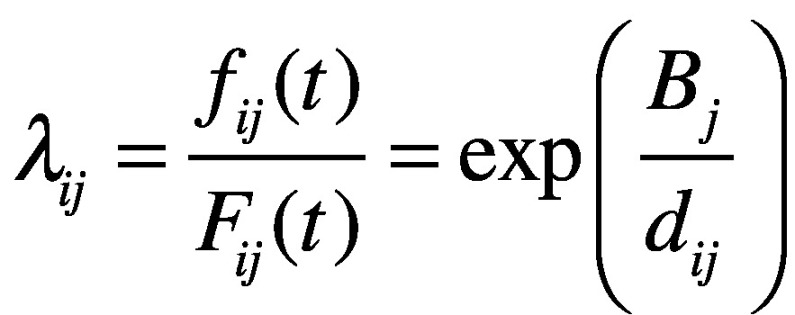
(2)where *β_j_* is a (country-specific) parameter that represents the risk of importing SARS-CoV-2. This formulation allows the median time of importation to be expressed as a function of the effective distance *d_ij_*. Using this hazard function, we model the probability density function of survival probability as:
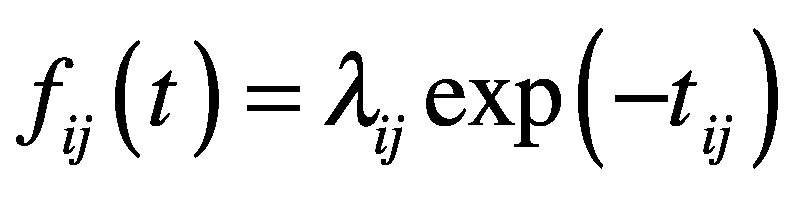
(3)where *t_ij_*is survival time at the *i*th airport in the *j*th country.

We categorized the 200 countries or territories included in the analysis into three separate groups: A, those that imported SARS-CoV-2 before travel restrictions were put in place; B, those that imported SARS-CoV-2 after the introduction of travel restrictions; and C, those that had not imported the virus as of the end of this study (26 February 2020).^10^^,^^18^ All category A,B and C countries or territories are listed in Table 1.

We define the likelihood of a country falling into category A, that is, the likelihood of a country importing the virus before the implementation of travel restrictions, as
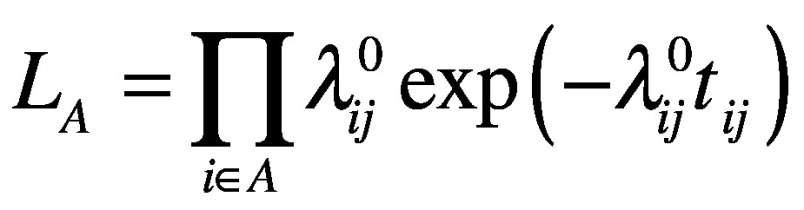
(4)where 
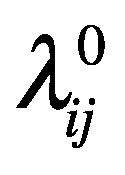
 represents the hazard function calculated according to the effective distance before the travel restriction, that is, 
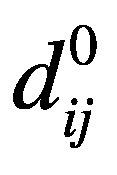
. Similarly, we define the likelihood of a country falling into category B as
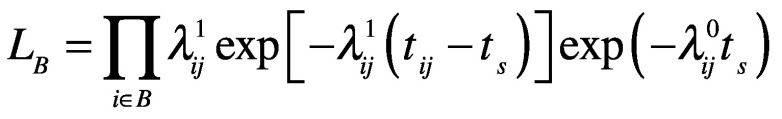
(5)where 
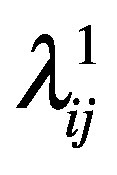
 indicates the hazard function calculated from the effective distance after the travel restriction 
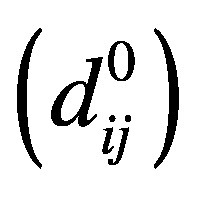
 and *t_s_* is the time between first reported case of the disease in Wuhan (8 December 2019) and the day on which the WHO described the outbreak as a Public Health Emergency of International Concern (30 January 2020, i.e. *t_s_* = 53 days). *L*_B_ is therefore the joint likelihood of avoiding the importation of the virus for *t_s_* days before travel restrictions and the probability of importing the virus during *t_ij_ − t_s_* (i.e. the survival time after 30 January 2020).

We define the likelihood of a country falling into category C as 
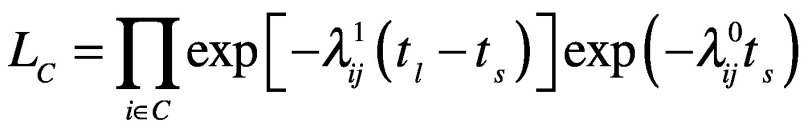
(6)where *t_l_*is the end of this study (26 February 2020). Equation 6 is the joint likelihood of the probability of avoiding the importation of SARS-CoV-2 for *t_s_* days before the travel restriction and for *t_l_ − t_s_* days after the travel restriction. 

Finally, we estimate the country-specific risk of importing SARS-CoV-2 by optimizing the product *L*_A_*L*_B_*L*_C_. We used a maximum likelihood approach to estimate *β_j_* and calculated 95% confidence intervals based on the empirical Fisher information matrix.

### Hypothetical scenarios

We calculated the effect of travel restrictions on the risk of virus importation by comparing the observed risk and that of three different hypothetical scenarios. Since the hazard is fixed over the time period, the cumulative risk of virus importation for the *i*th airport in the *j*th country was observed to be 
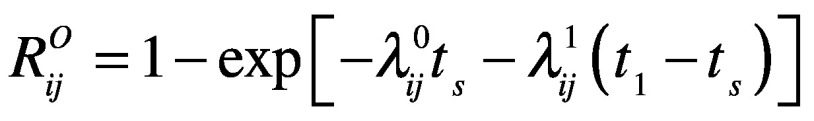
.

We considered three hypothetical scenarios that would all lead to different flight volumes (i.e. numbers of direct flights): H1, no travel restrictions had been introduced; H2, travel restrictions had been introduced, but only 25% or 50% of flights were cancelled instead of the current assumption of 75%; and H3, in addition to the travel restrictions introduced in the 80 countries listed in Table 1, we assumed that travel restrictions had also been introduced in the 10 highest-passenger-volume (i.e. hub) airports not included in Table 1 (namely Brussels (Belgium); Lester B. Pearson International, Toronto (Canada); Baiyun, Beijing and Shanghai (China); Dublin International (Ireland); Schipol, Amsterdam (Netherlands); Barajas, Madrid and Aeropuerto El Prat de Barcelona (Spain); Suvarnabhumi Bangkok International, Bangkok (Thailand), leading to a reduction in flight volume. We define the cumulative risk of virus importation under scenario H1 as 
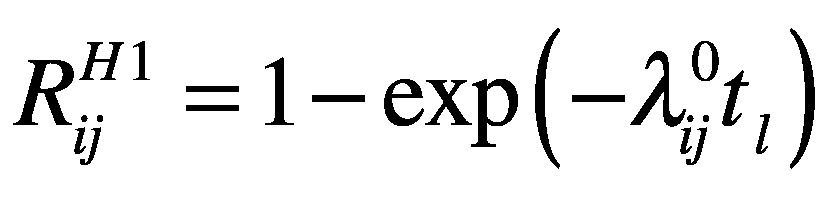
.

We define the cumulative risks of virus importation under scenarios H2 and H3 as 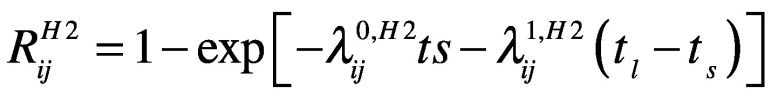 and 
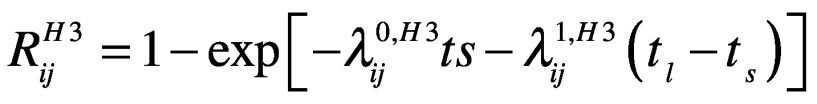
, where 
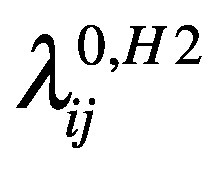
 and 
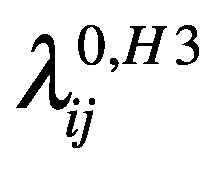
 are the hazard functions for scenarios H2 and H3, respectively. 

### Change in risk

To quantify the effect of travel restrictions on virus importation, we calculated the absolute and relative change in risk. The absolute change in risk is simply the difference between the observed risk and the risk incurred under a particular hypothetical scenario, defined as 
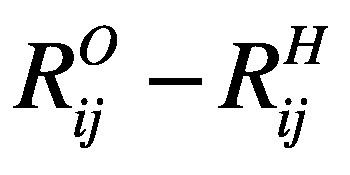
. The relative change in risk is the absolute change in risk under a particular hypothetical scenario expressed as a proportion of the observed risk, that is, 
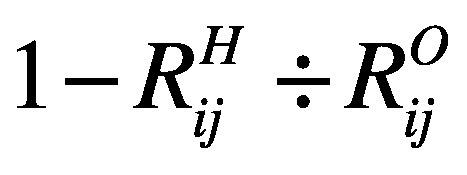
. A negative (positive) relative change in risk implies that the risk of virus importation under the hypothetical scenario would be higher (lower) than the observed risk.

## Results

We list the countries that introduced travel restriction policies and/or experienced importation of the virus in Table 1. A total of 28 countries had cases of COVID-19 as at 26 February 2020. A total of 21 of these countries imported the virus before implementing travel restrictions (category A) and seven countries imported the virus after the introduction of travel restrictions (category B). The arrival time of the virus ranged from 39 to 80 days since the first case was identified in Wuhan on 8 December 2019. 

**Fig. 1:**
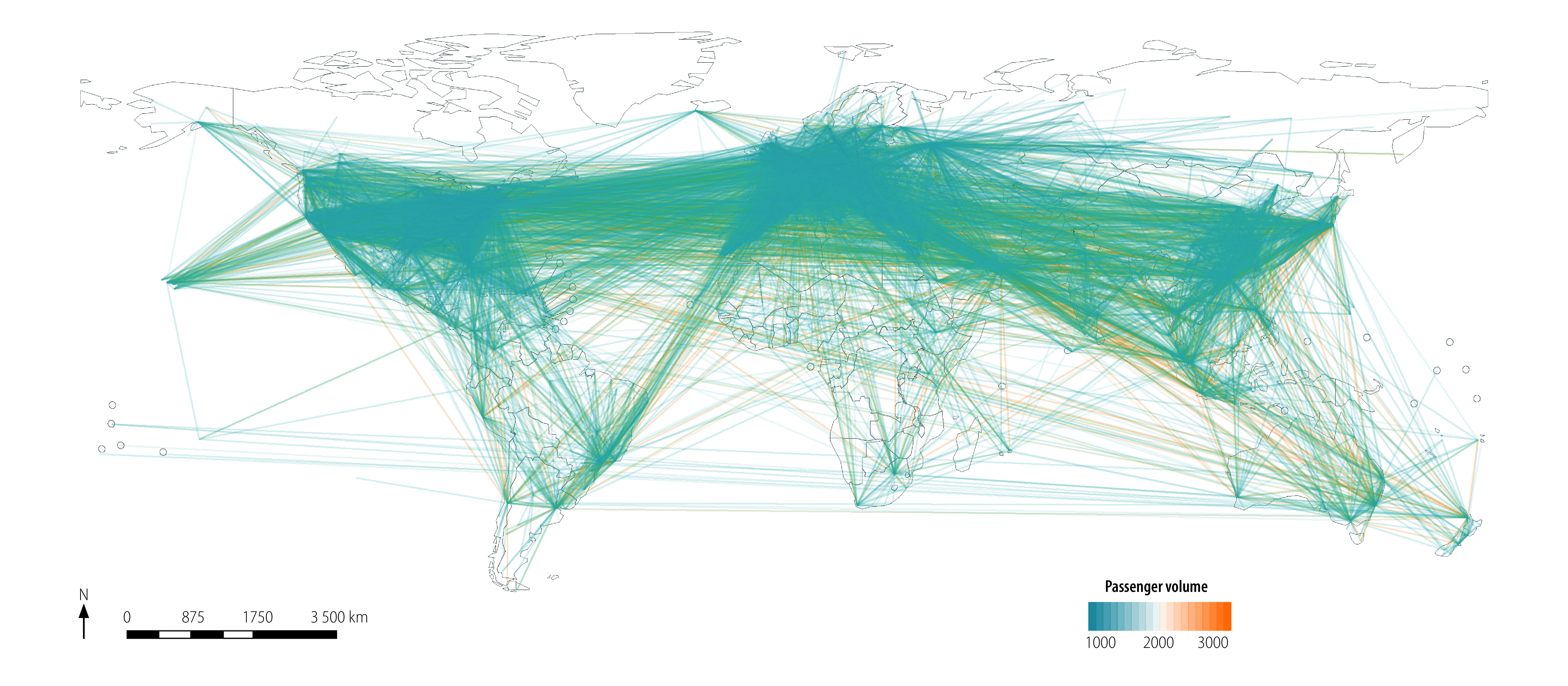
Entire global airline network diagram as at 1 December 2019

**Fig. 2:**
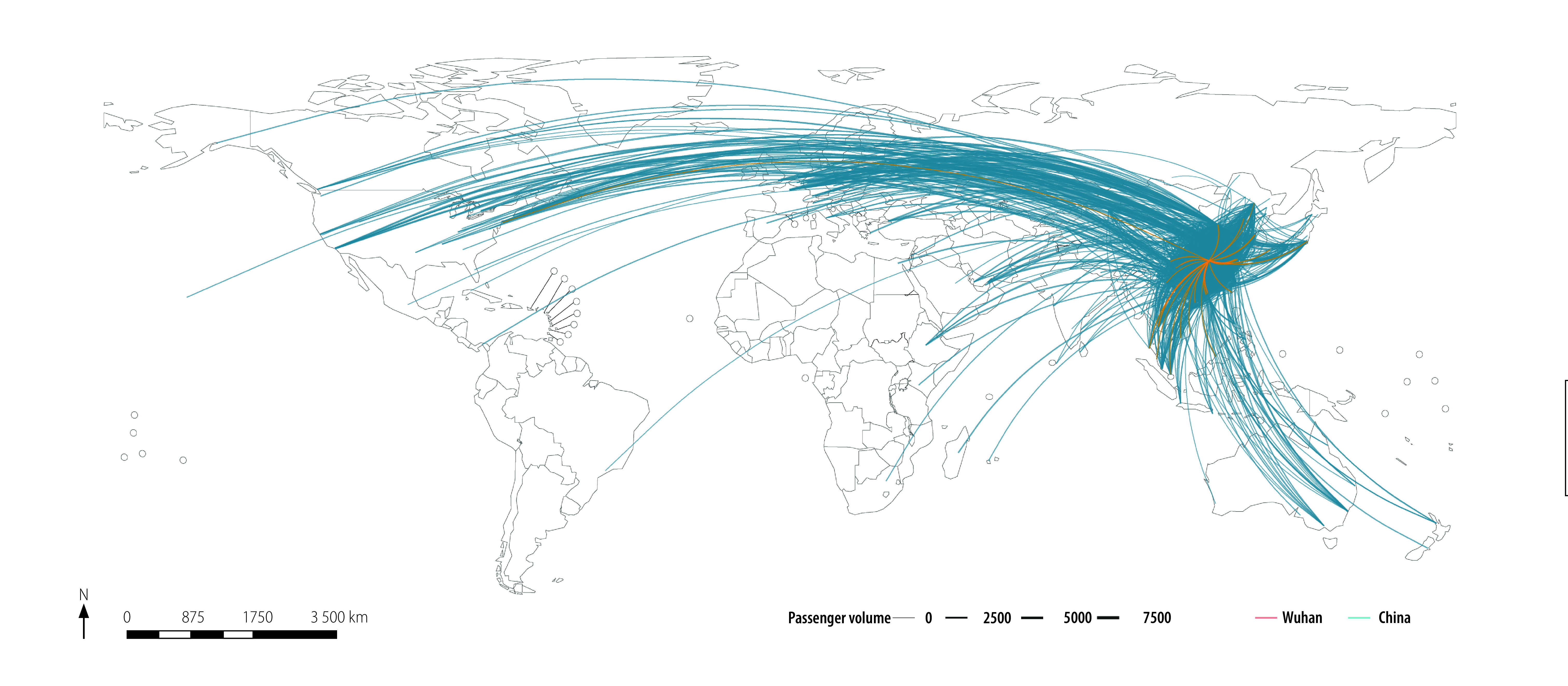
Airline network diagram for China and Wuhan city as at 1 December 2019

Fig. 1 and Fig. 2 depict the entire global airline network diagram and the flight network from all of China (as well as from Wuhan city only) before the introduction of travel restrictions, respectively.

**Fig. 3:**
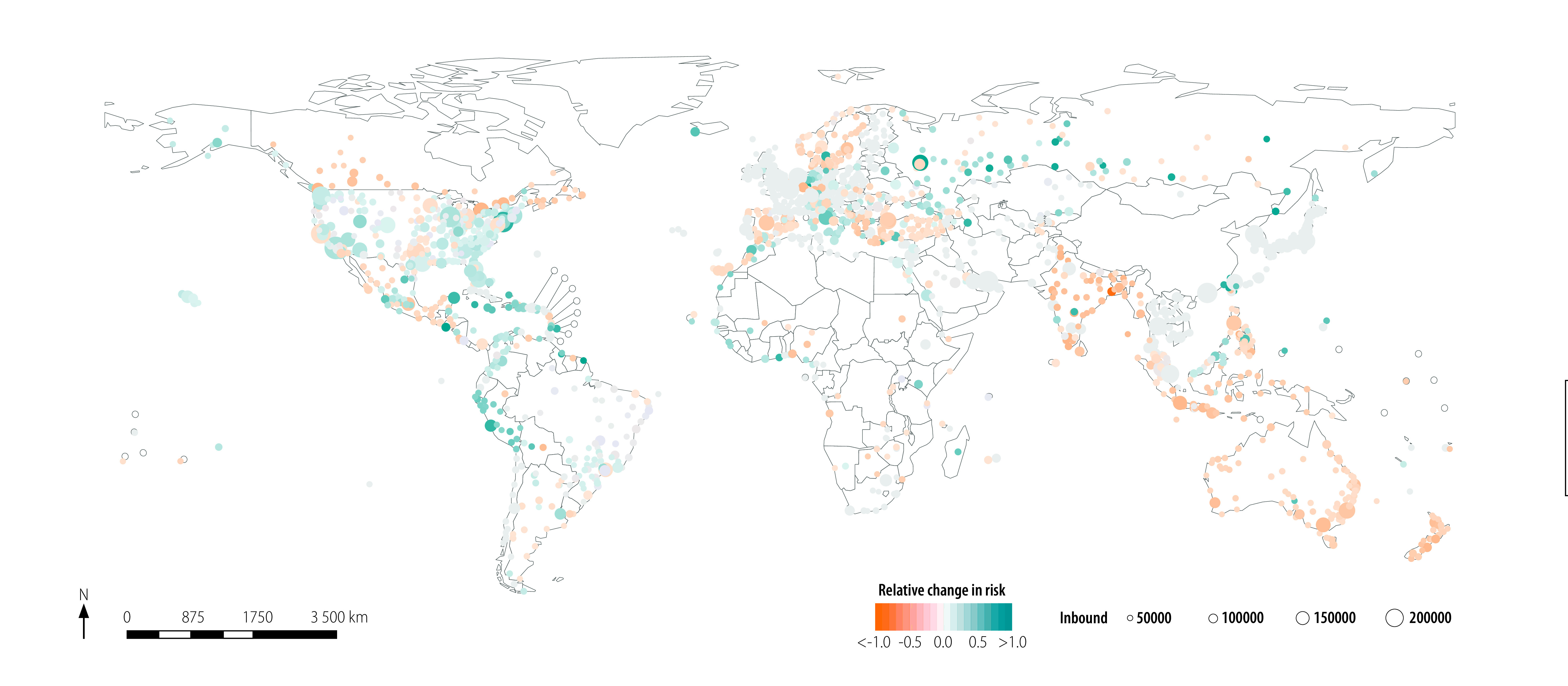
Estimated relative change in risk of SARS-CoV-2 importation under the hypothetical scenario that no travel restrictions had been introduced

Note: In the main text, this scenario is referred to as hypothetical scenario 1. Positive change in relative risk (green) equals lower risk of virus importation.

**Fig. 4:**
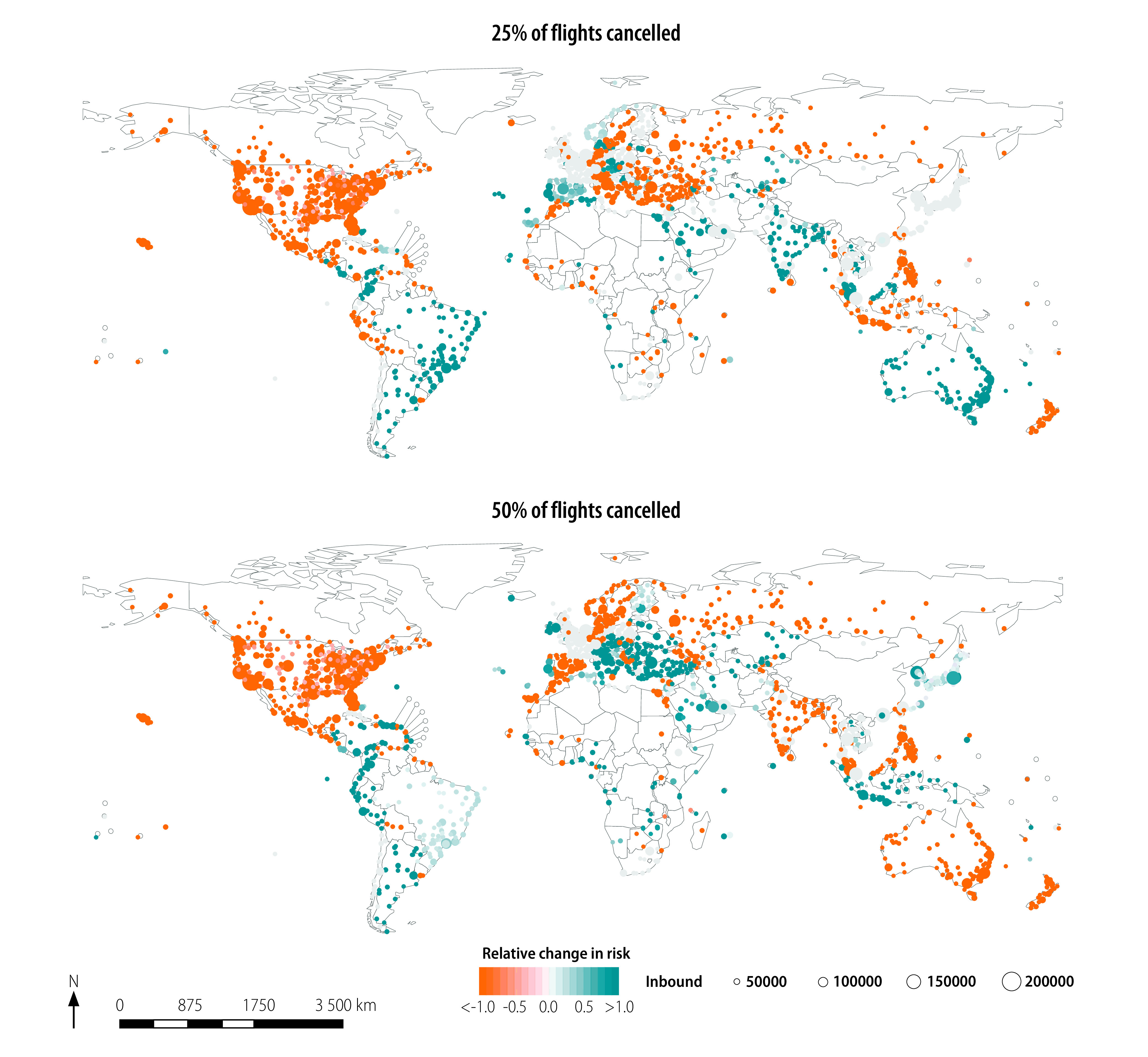
Estimated relative change in risk of SARS-CoV-2 importation under the hypothetical scenario that travel restrictions had been introduced, but only 25% or 50% of flights had been cancelled

Notes: In the main text, this scenario is referred to as hypothetical scenario 2. The original assumption was 75% of flights had been cancelled. Positive change in relative risk (green) equals lower risk of virus importation.

**Fig. 5:**
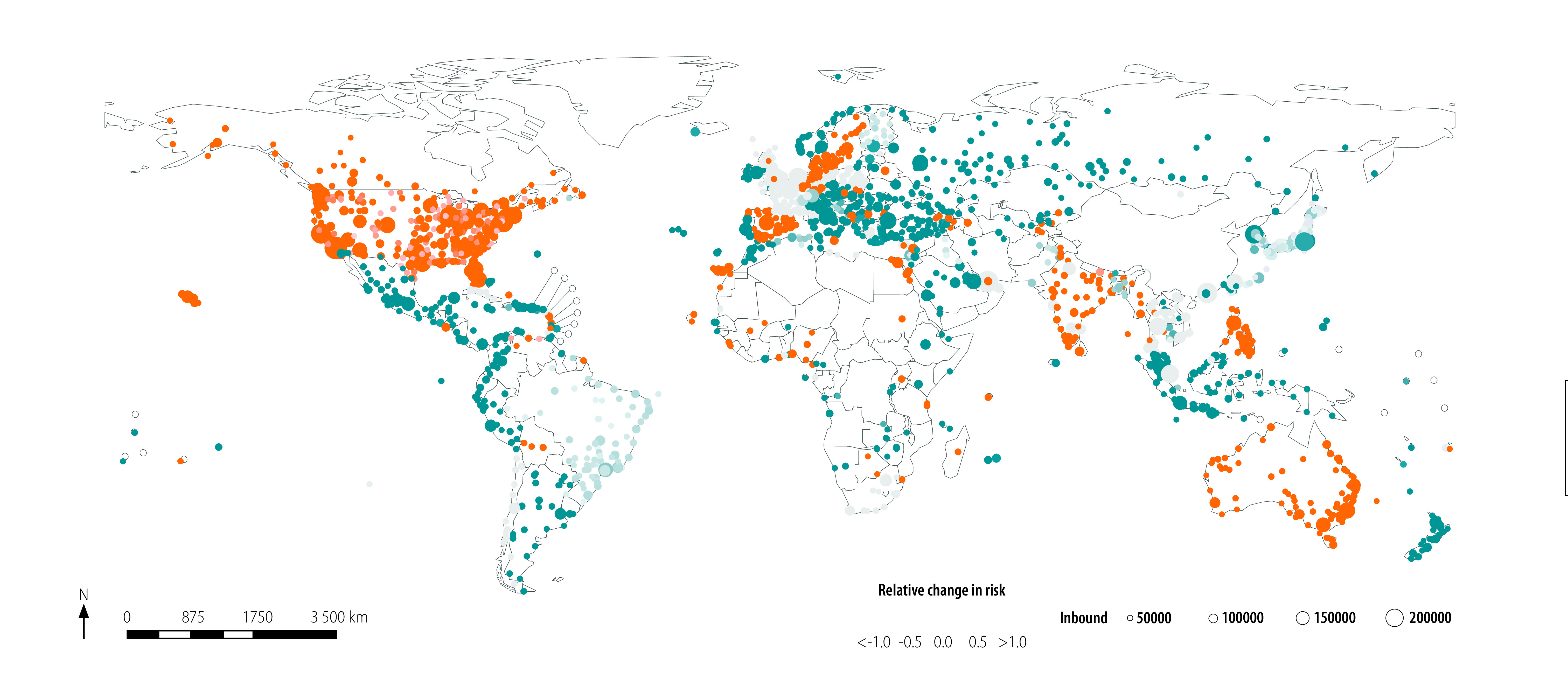
Estimated relative change in risk of SARS-CoV-2 importation under the hypothetical scenario that travel restrictions also had been introduced in the 10 high-passenger-volume airports

Notes: In addition to the travel restrictions introduced in the 80 countries listed in Table 1, we assumed that travel restrictions had also been introduced in the 10 highest-passenger-volume airports not included in Table 1. That is, Brussels (Belgium); Lester B. Pearson International, Toronto (Canada); Baiyun, Beijing and Shanghai (China); Dublin International (Ireland); Schipol, Amsterdam (Netherlands); Barajas, Madrid and Aeropuerto El Prat de Barcelona (Spain); Suvarnabhumi Bangkok International, Bangkok (Thailand). Positive change in relative risk (green) equals lower risk of virus importation.

We plot the estimated relative change in risk of importing the virus, because of the three different hypothetical travel restrictions and associated changes in effective distance, in Fig. 3, Fig. 4 and Fig. 5, respectively. The median relative change in risk and the corresponding interquartile range for all countries and territories, and three hypothetical scenarios analysed are available in the data repository.^17^

We plot the relative change in risk of virus importation under hypothetical scenario 1 in Fig. 3. We modelled the median (25% and 75% percentiles) of the estimated change in absolute and relative risk as 0.000 (0.000 and 0.000) and 0.000 (−0.115 and 0.049), respectively. When we reduced the global passenger volume between China and other airports as defined in hypothetical scenario 1, we observed a positive relative change in risk (i.e. lower risk of virus importation) in the coastal areas of Africa and South America, as well as in Europe and South-East Asia. 

Fig. 4 shows the change in relative risk under hypothetical scenario 2. We calculated the median (25% and 75% percentiles) of the estimated change in absolute and relative risk as −0.000 (−0.045 and 0.000) and −1.000 (−7.400 and 0.000) for only 25% flight cancellation and 0.000 (−0.039 and 0.000) and 0.000 (−3.000 and 1.000) for only 50% flight cancellation, respectively. Of the total number of airports considered, 45.5% (757/1662) and 44.6% (742/1662) showed large increases in risk (i.e. relative change in risk of < −1.0) for only 25% and 50% of flights cancelled, respectively. Note that the geographical distribution of the relative change in risk in Fig. 4 is similar to that in Fig. 3, except for the positive change in relative risk (i.e. lower risk of virus importation) under the hypothetical scenario 2 where 25% of flight has been cancelled in Australia and central Europe in Fig. 4. 

We modelled the estimated absolute and relative change in risk of virus importation under the assumption of hypothetical scenario 3 (Fig. 5) as 0.000 (−0.394 and 0.002) and 0.000 (−3.000 and 1.000), respectively. The results under these assumptions are geographically similar to those under hypothetical scenario 1 (Fig. 3); by introducing travel restrictions at the next 10 highest-passenger-volume hub airports, airports in coastal Africa and South America and all of Europe and South-East Asia could observe a reduction in risk of virus importation.

## Discussion

We have shown that the impact of travel restrictions was limited for most airports, with almost zero (median) change in risk of virus importation. The degree of travel restriction was assumed to be a 75% reduction in the flight volume. As a result, almost all airports would have observed a minor relative change in risk. To investigate the effect of passenger volume on the relative change in risk, we changed the passenger volume reduction from 75% to 50% or 25%. We observed a volume-dependent increase in risk in several areas including North America, part of Europe and the Russian Federation. Notably, when evaluating the effect of cancelling only 25% or 50% of flights (as opposed to 75% in the original assumption), the overall geographical distribution of the relative change in risk was similar, suggesting that passenger volume has a nonlinear effect on risk and the optimal volume reduction may depend on the particular airport and its network. From our results, we can conclude that travel restrictions based on reductions in passenger volume would only make a minor contribution to the prevention of virus importation among countries.

To confirm the expected reduced risk because of imposing travel restrictions at more hub airports, we estimated the relative change in risk under such an assumption. Notably, this result may be equivalent to evaluation of the effect of imposing lockdown in a country close to China, as hypothetical scenario 3 assesses the effect of closing three airports in China with direct flights from Wuhan and seven hub airports from six other countries. We observed that almost all airports experienced a decreased risk of virus importation under this scenario. However, Australia, India and the United States showed an increased risk of virus importation. A possible explanation for this result is that imposing travel restrictions at hub airports increases the number of passengers at other airports; this in turn changes the relative importance of specific airports, meaning that other airports could achieve hub airport status. These results suggest that careful consideration of which hub airports to restrict is essential.

Our study had limitations. First, as in previous studies,^10^^,^^21^ the infected individual was assumed to be randomly selected from the source of the virus (i.e. China). If infected individuals had particular characteristics, such as a preference for a particular route from China to other destinations (e.g. according to cost or visa restrictions), our results may be biased. Second, we did not control for any covariates (e.g. culture, religion or country income level), something which should be addressed in future studies.^10^

Air travel is not the only force driving the spread of the virus. In countries sharing a land border or separated by a small stretch of water, the effect of sea and land travel on the spread of the virus could be greater than that of air travel; we therefore propose further research in calculating the effective distance between locations based on the transportation data from air, sea and land travel. 

Although strategies to restrict the airline network may reduce the risk of SARS-CoV-2 importation at certain risk-sensitive airports, restrictions will also have significant economic and social impacts.^22^ Restricting the airline network to control an emerging infectious disease therefore requires global collaboration; a framework in which country representatives and experts work closely together to calculate science-based risks and consider travel restrictions at relevant airports will be essential. It should also be recognized that most countries must strengthen their local capacity for disease monitoring and control, rather than attempting to reduce the risk of virus importation via airline networks. 
