# Corrigendum

**DOI:** 10.2471/BLT.20.110920

**Published:** 2020-09-01

In: Shoi Shi, Shiori Tanaka, Ryo Ueno, Stuart Gilmour, Yuta Tanoue, et al. Travel restrictions and SARS-CoV-2 transmission: an effective distance approach to estimate impact. Bull World Health Organ. 2020 Aug 1;98(8):518–529 On page 521, equation 3 should read as follows: 


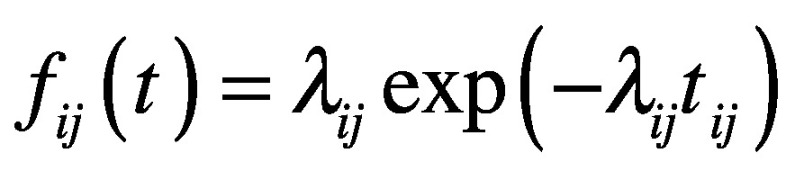
(3)

